# Selection based on morphological features of porcine embryos produced by in vitro fertilization: Timing of early cleavages and the effect of polyspermy

**DOI:** 10.1111/asj.13401

**Published:** 2020-06-11

**Authors:** Hiep Thi Nguyen, Thanh Quang Dang‐Nguyen, Tamas Somfai, Nguyen Thi Men, Nguyen Viet Linh, Bui Xuan Nguyen, Junko Noguchi, Hiroyuki Kaneko, Kazuhiro Kikuchi

**Affiliations:** ^1^ Institute of Agrobiological Sciences National Agriculture and Food Research Organization (NARO) Tsukuba Japan; ^2^ The United Graduate School of Veterinary Science Yamaguchi University Yamaguchi Japan; ^3^ Institute of Biotechnology Vietnam Academy of Science and Technology Hanoi Vietnam; ^4^ Institute of Livestock and Grassland Science NARO Tsukuba Japan

**Keywords:** abnormalities, chromosome, nucleus, polyspermy, porcine embryos

## Abstract

The aim of this study was to examine whether a morphological approach is efficient for selecting high‐quality porcine embryos produced by in vitro fertilization (IVF) under high polyspermy conditions. Frozen‐thawed Meishan epididymal spermatozoa showing moderate and high polyspermy were subjected to IVF (1 × 10^5^ sperms/ml). Under conditions of moderate polyspermy, 4‐cell embryos selected at 48 hr after IVF (single selection) and 8‐cell embryos selected at 79 hr after IVF from the collected 4‐cell embryos (double selection) showed high developmental competence. Likewise, 4‐ and 8‐cell embryos produced by IVF under high polyspermy conditions also showed high competence for development to blastocysts. However, blastocysts derived from high polyspermy conditions had significantly fewer cells than those produced under moderate polyspermy conditions. Furthermore, the frequency of nuclear and chromosomal abnormalities in 4‐ and 8‐cell embryos produced under conditions of high polyspermy was significantly (*p* < .05) higher in comparison to moderate polyspermy conditions. These findings suggest that although high polyspermy affects the frequency of nuclear and chromosomal anomalies in porcine IVF embryos, subsequent selection based on morphological features of 4‐ and 8‐cell embryos even under high polyspermy conditions, could be an alternative option for selecting porcine IVF embryos with high development ability.

## 1 INTRODUCTION

In vitro production (IVP) of porcine embryos is an important tool for porcine gene banking (Kikuchi et al., 2016) as well as for human biomedical research (Niemann & Rath, 2001). However, in vitro fertilization (IVF) in pigs is associated with a high frequency of polyspermy, causing chromosomal abnormalities in embryos (Abeydeera & Day, 1997; Herrick, Conover‐Sparman, & Krisher, 2003; Koo et al., 2005; Nagai, Funahashi, Yoshioka, & Kikuchi, 2006). Polyspermy also occurs during IVF in humans (Kola, Trounson, Dawson, & Rogers, 1987; Rudak, Dor, Mashiach, Nebel, & Goldman, 1984) and cattle (Iwasaki et al., 1992; Iwasaki, Shioya, Masuda, Hanada, & Nakahara, 1989) but at lower frequency. Although various approaches have been tried for reduction in polyspermy in pigs, production of normal porcine embryos in vitro remains a challenge (Grupen, 2014; Nagai et al., 2006).

Although IVP embryos are able to develop to term after embryo transfer (ET) (Kikuchi et al., 2002; Yoshida, Mizoguchi, Ishigaki, Kojima, & Nagai, 1993), their quality is still lower than those of embryos produced in vivo (Han, Abeydeera, et al., 1999; Kikuchi, Kashiwazaki, Noguchi, et al., 1999; McCauley et al., 2003). A higher incidence of chromosomal abnormalities has been reported in IVP embryos relative to their in vivo counterparts, especially in pigs (McCauley et al., 2003; Ulloa Ulloa, Yoshizawa, Komoriya, et al., 2008; van der Hoeven, Cuijpers, & de Boer, 1985). Although some polyspermic zygotes have been reported to develop to piglets (Han, Wang, et al., 1999), most embryos with chromosomal abnormalities fail to develop to term (Plachot, 1989; Causio, Fischetto, Sarcina, Geusa, & Tartagni, 2002, reviewed in Yoshizawa, 2003). Therefore, for ET, it is important to select embryos with a low frequency of chromosomal abnormalities using simple and reliable procedures.

In humans and many other mammalian species it has been shown that the developmental competence of embryos is linked with the timing of early cleavages after IVF (Alikani et al., 2000; Dang‐Nguyen et al., 2010; Edirisinghe et al., 1992; Lonergan et al., 1999; Magli et al., 2007; McKiernan & Bavister, 1994; Ulloa Ulloa, Yoshizawa, Komoriya, et al., 2008; Ulloa Ulloa, Yoshizawa, Yamashita, et al., 2008). Those previous studies showed that good‐quality embryos could be selected based on their morphological features and the timing of early cleavages. Bovine embryos at the 5‐ to 8‐cell stage selected on Day 2 (Day 0 = IVF) had lower incidences of chromosomal abnormalities (Ulloa Ulloa, Yoshizawa, Yamashita, et al., 2008). Similarly, porcine embryos at the 3‐ to 4‐cell stage and the 5‐ to 8‐cell stage selected 52 hr after IVF (Ulloa Ulloa, Yoshizawa, Komoriya, et al., 2008), or 2‐cell stage embryos selected 30 hr after IVF (Dang‐Nguyen et al., 2010) were proven to have lower incidences of chromosomal abnormalities and high developmental competence. Other studies have also shown that the timing of cleavage, evenness of division, and the degree of fragmentation can also be useful criteria for predicting the blastocyst formation ability of early porcine embryos (Booth, Watson, & Leese, 2007; Dang‐Nguyen et al., 2010; Mateusen et al., 2005). However, whether or not such morphological evaluation could also be effective for selecting high‐quality embryos under high polyspermy conditions has not been investigated.

In a preliminary experiment using our current IVP system, with the support of time‐lapse cinematography, we found that single selection of 4‐cell embryos and double selection of 8‐cell embryos showed a high potential for rapid development to good‐quality blastocysts (Nguyen et al., unpublished). Consequently, the present study was conducted to clarify whether selection of 4‐ and 8‐cell stage embryos as single‐ and double‐selected embryos at specific time points might be effective for ensuring good embryo quality, and the degree to which a high rate of polyspermy might affect the developmental competence, nuclear status, and karyotype of the selected embryos. It was considered that the data from this study would lead to an alternative option for selection of embryos at fixed time points based on their morphological features, and furthermore yield insight into whether a selection approach based on embryonic morphology would still be effective under high polyspermy conditions, an aspect that is essential for porcine reproduction studies where a high polyspermy rate is often unavoidable.

## 2 MATERIALS AND METHODS

### 2.1 Reagents

All reagents were obtained from Sigma‐Aldrich Chemical Co. unless otherwise stated.

### 2.2 Oocyte collection and in vitro maturation (IVM)

The ovaries were collected from prepubertal cross‐bred gilts (Landrace × Large White × Duroc) at a local slaughterhouse, and carried to the laboratory in Dulbecco's phosphate‐buffered saline (PBS; Nissui Pharmaceutical Co. Ltd.) at 35–37°C within 1 hr. Cumulus–oocyte complexes (COCs) were collected from follicles 3–6 mm in diameter in collection medium consisting of Medium 199 (with Hanks’ salts) supplemented with 10% fetal bovine serum (Gibco; Thermo Scientific), 20 mM HEPES (Dojindo Laboratories), and antibiotics (100 units/ml penicillin G potassium and 0.1 mg/ml streptomycin sulfate). IVM of oocytes was carried out as reported previously (Kikuchi, Kashiwazaki, Noguchi, et al., 1999). In brief, about 50 COCs were cultured in each 500 µl of maturation medium, a modified North Carolina State University (NCSU)‐37 solution (Petters & Wells, 1993) containing 10% (v/v) porcine follicular fluid, 0.6 mM cysteine, 50 mM β‐mercaptoethanol, 1 mM dibutyryl cyclic adenosine 3ʹ,5ʹ‐monophosphate (dbcAMP), 10 IU/ml equine chorionic gonadotropin (Serotropin; ASKA Pharmaceutical Co. Ltd.), and 10 IU/ml human chorionic gonadotropin (Gonatropin; ASKA) in four‐well dishes (Nunclon Multidishes, Nunc; Thermo Fisher Scientific) for 22 hr in an atmosphere of 5% CO_2_, 5% O_2_, and 90% N_2_ at 38.5°C. The COCs were subsequently cultured in the maturation medium without dbcAMP and hormones for an additional 24 hr under the same atmosphere.

### 2.3 Preparation of epididymal spermatozoa

Epididymal spermatozoa were collected and frozen according to Kikuchi et al. (1998), Kikuchi, Kashiwazaki, Nagai, et al. (1999). Briefly, epididymides from four Meishan boars were brought to the laboratory just after the slaughter at Institute of Livestock and Grassland Science, NARO (NILGS) at room temperature. Luminal fluid containing spermatozoa was extruded from the distal portion of the cauda epididymidis by air pressure using a syringe. The fluid was diluted with 30 ml of collection solution at room temperature. The sperm suspension was then cooled to 15°C over about 3 hr. The solution containing the spermatozoa was centrifuged at 1,200 *g* in for 10 min at 4°C and the supernatant was discarded. The precipitated spermatozoa were gently resuspended with Niwa and Sasaki Freezing (NSF)‐I extender at 4°C (Niwa, 1989), then diluted with the same volume of NSF‐II at the same temperature. The concentrations of sperm were diluted to 1 × 10^9^ (Boar A) or 5 × 10^8^ sperm/ml (Boars B–D) before freezing. Whereas, motility after collection was higher than 80% in all the four examined boars. The sperm suspension was then transferred into 0.25‐ml plastic straws (IMV, L’Aigle), which were placed in liquid nitrogen vapor for 10 min and finally stored in liquid nitrogen.

### 2.4 IVF and in vitro culture (IVC)

COCs were treated with 0.1% (w/v) hyaluronidase to remove part of the cumulus using a glass pipet. IVF was performed according to the 2‐step IVF method of Grupen and Nottle (2000) with some modifications. The medium used for IVF was a modified Pig‐FM medium (Suzuki et al., 2002) containing 10 mM HEPES, 2 mM caffeine, and 5 mg/ml bovine serum albumin (BSA). The oocytes were washed three times in IVF medium. They were then transferred into 90‐µl IVF droplets (approximately 20 oocytes in each droplet) covered with paraffin oil (Paraffin Liquid; Nacalai Tesque). Frozen‐thawed epididymal spermatozoa from each of Meishan boars was preincubated at 38.5°C in Medium 199 (with Earle's salts, Gibco, pH adjusted to 7.8) for 15 min (Ikeda et al., 2002; Kikuchi et al., 1998). Matured oocytes were co‐incubated with preincubated sperm at 1 × 10^5^ sperms/ml (Ikeda et al., 2002; Kikuchi et al., 2002) for 30 min at 38.5°C under 5% CO_2_, 5% O_2_, and 90% N_2_. The oocytes with the zona‐bound sperm were then transferred to other fresh droplets of the IVF medium and subsequently incubated for 2.5 hr. At the end of IVF, spermatozoa were removed from the surface of the zona pellucida by gentle pipetting with a fine‐glass pipette. The day of IVF was defined as Day 0. The basic IVC medium was NCSU‐37 medium containing 4 mg/ml BSA and 50 mM β‐mercaptoethanol. The putative zygotes were cultured in 500 µl drops of IVC‐PyrLac, basic medium supplemented with 0.17 mM sodium pyruvate and 2.73 mM sodium lactate, for Days 0–2 and in IVC‐Glu, basic medium with 5.55 mM D‐glucose (Wako Pure Chemical Industries, Ltd.) and for Days 2–6 (Kikuchi et al., 2002) in four‐well dishes in an atmosphere of 5% CO_2_, 5% O_2_ and 90% N_2_ at 39°C.

### 2.5 Polyspermy evaluation

**FIGURE 1:**
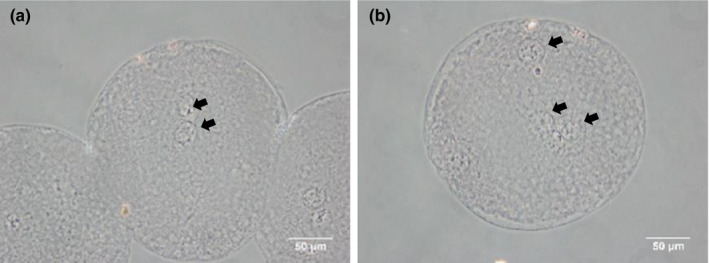
Pronucleus(ei) in zygotes at 10 hr after IVF. A monospermic (a) and a polyspermic (b) zygote. Arrows show pronucleus(ei)

Putative zygotes were fixed at 10 hr after IVF in a mixture of acetic acid and absolute ethanol (1:3) for at least 3 days, stained with 1% aceto‐orcein, and examined for sperm penetration and male pronucleus formation (MPN) under a phase‐contrast microscope. Zygotes with one female pronucleus and a single sperm head or MPN in the cytoplasm were considered to be monospermic (Figure 1a). Zygotes with more than one sperm and/or MPNs were considered to be polyspermic (Figure 1b). In the present study, polyspermy rates of approximately 60% or lower and 90% or higher were considered as "moderate" and "high" polyspermy conditions, respectively.

### 2.6 Embryo evaluation

An embryo with more than 10 cells and a clear blastocoel was defined as a blastocyst. The rate of blastocyst formation and the total number of cells per blastocyst were examined on Day 6. The blastocysts were fixed and stained in ethanol containing 25 μg/ml Hoechst 33,342 (Calbiochem; EMD Biosciences Inc.) and examined under an epifluorescence microscope (Olympus). The total number of cells per blastocyst was evaluated as an indicator of embryo quality.

### 2.7 Nuclear status evaluation

**FIGURE 2:**
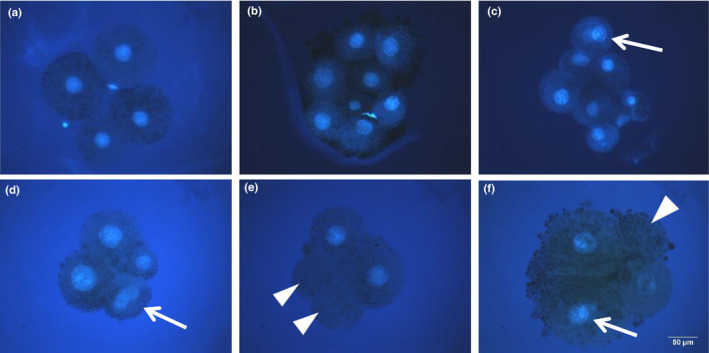
Nuclear staining of 4‐ and 8‐cell stage embryos. Normal embryos at the 4‐cell stage (a) and 8‐cell stage (b). Nuclear abnormalities in 4‐ and 8‐cell stage embryos: an 8‐cell embryo carrying a binuclear blastomere (c), a 4‐cell embryo with a binuclear blastomere (d), a 4‐cell embryo containing two anuclear blastomeres (e), and 4‐cell embryos with a binuclear and an anuclear blastomeres (f). Arrows show binuclear blastomeres and arrowheads show anuclear blastomeres

The zona pellucida was removed single‐ and double‐selected embryos using 1% pronase (protease, P8811). They were then fixed in 4% paraformaldehyde (PFA) for at least 3 hr, stained with 4′,6‐diamidino‐2‐phenylindole (DAPI) in a mounting medium (Vectashield; Vector Laboratories Inc.), and visualized using an epifluorescence microscope (Olympus). Embryos in which all blastomeres contained exactly one nucleus were considered normal (Figure 2a,b). Embryos with one or several blastomere(s) carrying no or more than one nucleus(ei) were considered abnormal (Figure 2c–f).

### 2.8 Chromosome analysis

**FIGURE 3:**
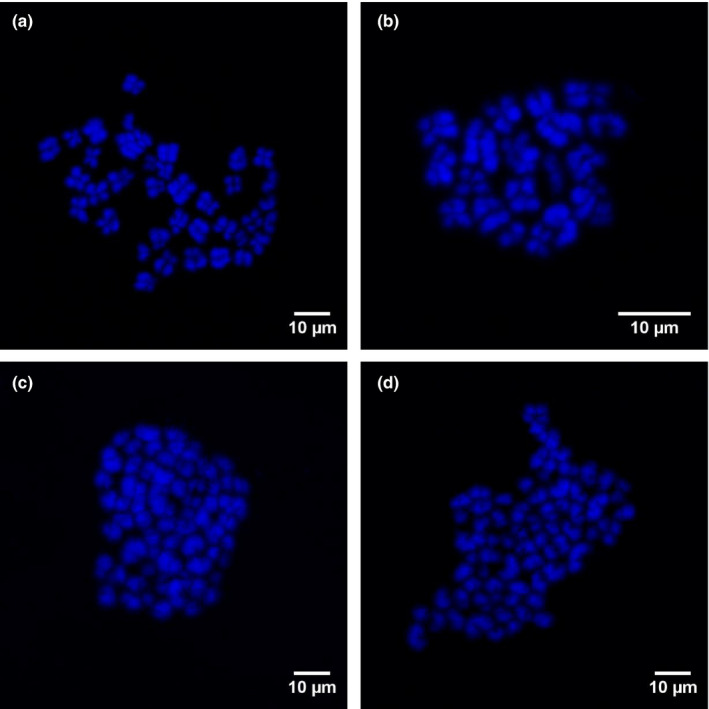
Blastomere ploidy in porcine embryos. Metaphase spread from a diploid (2n) cell with two chromosome sets (a). Ploidy abnormalities: haploid (n; b), triploid (3n; c), and tetraploid (4n; d)

Chromosome samples from single‐ and double‐selected embryos, respectively, were prepared according to Yoshizawa et al. (1998) with some modifications. Briefly, the single‐ and double‐selected embryos were treated with 20 ng/ml colcemid in IVC Glu for 17–20 hr. The embryos were then washed and incubated in 0.4 ml of 1% (w/v) hypotonic sodium citrate solution for 15 min and fixed mildly by adding 0.02 ml of acetic acid: methanol (1:1) to the sodium citrate solution for 2 min. A single embryo was placed on a glass slide with a minimal volume of hypotonic solution, immediately covered with a very small droplet of acetic acid to separate the cells, and then refixed with several drops of acetic acid: ethanol (1:3). After being allowed to dry completely, chromosome samples were stained with DAPI in Vectashield and then visualized under an epifluorescence microscope (Olympus). Only embryos containing at least two well‐spread metaphases plates (intact and nonoverlapping) were analyzed. Embryos that had two sets of chromosomes (2n = 38) in all analyzable metaphase plates were defined as being diploid (Figure 3a), whereas those with only one set of chromosomes (*n* = 19) in all analyzable metaphase plates were defined as being haploid (Figure 3b). Embryos containing more than two sets of chromosomes in all countable metaphase plates were defined as being polyploid (3n, 4n, etc.) (Figure 3c, d). Embryos containing a mixture of diploid and haploid cells (n/2n), triploid (2n/3n), tetraploid (n/4n), or other types of polyploid cells were defined as being mixoploid.

### 2.9 Experimental design

#### 2.9.1 Experiment 1

We first aimed to establish moderate and high polyspermy conditions. Firstly, four Meishan boars (A–D) has been examined to select "moderate" and "high" for the purpose of the present study. In our system, sperm acceptable for reasonable in vitro embryo production show polyspermy rate is 50%–100% and the penetration rate is 80%–100%. The low polyspermy rate could be obtained when reducing sperm concentration. However, it also means low penetration rate, and therefore resulting in low embryonic development. In the present study, the polyspermy rates at 50%–70% and >80% to be consider as "moderate" and "high" polyspermy rate, respectively.

#### 2.9.2 Experiment 2

In order to compare the developmental competence of embryos produced under moderate and high polyspermy conditions (evaluated and selected in Experiment 1), in vitro matured oocytes were fertilized with each type of sperm. Blastocyst formation rates and the numbers of cells per blastocyst were recorded after 6 days of IVC. The experiment was replicated six times.

#### 2.9.3 Experiment 3

In order to compare the effect of selection on developmental competence, three embryo groups were set. We have separated 4‐cell embryos and the other "remaining" cleaved (2‐ to 3‐cell) embryos at 48 hr after in vitro fertilization under moderate and high polyspermy conditions (as evaluated in Experiment 2). Both groups of embryos were cultured separately. Subsequently, from a group of the 4‐cell embryos, 8‐cell embryos as "double selected" embryos and the other cleaved (4‐ to 7‐cell) embryos were reseparated at 79 hr after IVF. Those embryos, as well as "remaining" embryos, were cultured until Day 6 after IVF. In this experiment, data of double‐selected and the other cleaved embryos are pooled and analyzed as "single‐selected" embryos. Blastocyst formation rates calculated from the selected embryos and cell numbers per blastocyst in the respective groups (single‐selected, double‐selected, and remaining embryos) were analyzed. Three to six replications with each sperm line were carried out.

#### 2.9.4 Experiment 4

In order to compare nuclear abnormalities of single‐ and double‐selected embryos produced under moderate and high polyspermy conditions (evaluated in Experiment 3), the oocytes after IVM were fertilized with each type of sperm. The single‐ and double‐selected embryos were subjected to nuclear staining to evaluate nuclear abnormalities as described above. Three to five replications with each sperm line were carried out.

#### 2.9.5 Experiment 5

In order to compare chromosomal abnormalities in single‐ and double‐selected embryos produced under moderate and high polyspermy conditions (evaluated in Experiment 3), in vitro–matured oocytes were fertilized with each type of sperm. The single‐ and double‐selected embryos were then used for chromosome spreads as described above. Three to six replications with each sperm line were carried out.

### 2.10 Statistical analysis

All data were expressed as mean percentage ±*SEM* values. The data were analyzed by one‐way ANOVA followed by Bonferroni correction by using the Stata/SE 15.0 software package (StataCorp.). Differences at *p* < .05 were considered statistically significant when it is not identified in the text.

## 3 RESULTS

### 3.1 Experiment 1

All four Meishan boars (A–D) were informed at the slaughter that they were over 1.5 years of age and used for this study. Penetration and polyspermy rates of these boar sperm lines were presented in Table 1. Zygotes derived from oocytes fertilized by Boar B sperm (90.4 ± 1.7%) had a significantly higher (*p* < .05) polyspermy rate than zygotes derived from oocytes fertilized by Boars D, C, and A sperm (80.3 ± 1.9%; 65.8 ± 4.3%; and 54.4 ± 6.9%, respectively; Table 1). In addition, the percentage of oocytes penetrated by Boar B sperm (97.1 ± 1.0%) was equal to that of Boar A sperm (83.8 ± 5.3%). As a result, sperm from Boars A and B were used for IVF to assess in the following experiments. Boar A was defined as “moderate” polyspermy rate, since the proportion of polyspermy produced from Boar A was lowest in the all samples. Boar B was selected to produce high polyspermy conditions.

**TABLE 1:** Penetration and polyspermy rates of different boar sperm lines

Boars	Total IVF	Penetration (% total)	Polyspermy (% penetration)
A	145	122 (83.8 ± 5.3)^a‐c^	69 (54.4 ± 6.9)^a‐c^
B	172	167 (97.1 ± 1.0)^a‐c^	151 (90.4 ± 1.7)^a‐c^
C	88	73 (83.0 ± 3.3)^a‐c^	48 (65.8 ± 4.3)^a‐c^
D	91	76 (83.5 ± 3.2)^a‐c^	61 (80.3 ± 1.9)^a‐c^

Note

Epididymal sperm were collected from Boars A to D.

Three to 10 replications of each sperm line were carried out.

Results are presented as mean percentage ± *SEM*.

^a‐c^ Values with different superscripts in the same column are significantly different (*p* < .05).

### 3.2 Experiment 2

The data for development of embryos produced under moderate and high polyspermy conditions are shown in Table 2. There were no differences in cleavage rate between embryos produced under moderate (77.9 ± 3.3%) and high (77.0 ± 3.0%) polyspermy conditions. However, the proportion of embryos that formed blastocysts and the total number of cells in blastocysts formed under moderate polyspermy conditions (28.6 ± 4.1% and 48.8 ± 1.8, respectively) were significantly higher (*p* < .05) than the corresponding figures for high polyspermy conditions (14.8 ± 2.7% and 38.5 ± 0.9, respectively).

**TABLE 2:** Development of porcine embryos produced by IVF under moderate and high polyspermy conditions

Polyspermy condition	No. of embryos	Cleavage (% IVF)	Blastocyst (% IVF)	Cell no./blastocyst
Moderate	582	446 (77.9 ± 3.3)	143 (28.6 ± 4.1)^a,b^	48.8 ± 1.8^a,b^
High	1,276	976 (77.0 ± 3.0)	218 (14.8 ± 2.7)^a,b^	38.5 ± 0.9^a,b^

Note

Sperm with moderate and high polyspermy were collected from Boars A and B, respectively.

Six replications of each sperm line were carried out.

Results are presented as mean percentage ± *SEM*.

^a,b^ Values with different superscripts in the same column are significantly different (*p* < .05).

### 3.3 Experiment 3

There were no differences in the numbers and percentages of selected 4‐cell embryos in total cleaved embryos produced under moderate (21.1 ± 1.0%) and high (17.0 ± 2.6%) polyspermy conditions (Table 3). However, the percentage of double selection under 8‐cell embryos produced under moderate polyspermy conditions (6.3 ± 0.6%) was significantly higher than that of selected 8‐cell embryos produced under high polyspermy conditions (3.4 ± 0.8%; Table 3).

**TABLE 3:** Comparison of developmental competence and cell number in porcine blastocysts derived from single‐, double‐selected and remaining embryos produced under moderate and high polyspermy conditions after IVF

Polyspermy condition	Total no. oocytes for IVF	No. cleaved embryos^$^	Embryo selection group	48 hr after IVF			79 hr after IVF			No. blastocysts (% of selected embryos)	Cell no./blastocyst
				2–3‐cell	4‐cell	4‐cell (% cleaved)	4–7‐cell	8‐cell	8‐cell (% cleaved)		
Moderate	1,045	769	Remaining	606			NA	NA		144 (24.0 ± 5.6)^a,b^	45.1 ± 2.2^a,b^
			Single*		163	21.1 ± 1.0	115^†^	48^#^		80 (50.2 ± 6.6)^*^	60.9 ± 2.5^*^
			Double					48	6.3 ± 0.6^A,B^	35 (70.7 ± 11.8)^c^	63.5 ± 2.9^A,B^
High	1,074	824	Remaining	687			NA	NA		137 (19.3 ± 4.4) ^a,b^	37.5 ± 1.3^A,B^
			Single*		137	17.0 ± 2.6	110^†^	27^#^		59 (40.8 ± 9.1)^*^	36.9 ± 1.6^A,B^
			Double					27	3.4 ± 0.8^A,B^	16 (57.9 ± 4.1)^c^	37.7 ± 3.0^A,B^

Note

Sperm with moderate and high polyspermy were collected from Boars A and B, respectively.

Three to four replications with each sperm line were carried out.

Results are presented as mean percentage ± *SEM*. NA, non applicable.

^$^ Mono cell embryos were discarded at 48 hr after IVF.

* Data of single‐selected embryos pooled from both double‐selected^#^ and other cleaved^†^ embryos separated at 79 hr after IVF.

^a,b^ Values with different superscripts are significantly different among embryo selection categories in the same polyspermy condition (*p* < .05).

^A,B^ Values with different superscripts are significantly different between polyspermy conditions in the same embryo selection group (*p* < .05).

Table 3 shows the developmental competence of single‐, double‐selected, and remaining embryos derived under moderate and high polyspermy conditions. Both sets of embryos showed a high potential for development to blastocysts under both moderate (50.2 ± 6.6% and 70.7 ± 11.8%, respectively) and high (40.8 ± 9.1% and 57.9 ± 4.1%, respectively) polyspermy conditions, and no significant difference was evident between them (Table 3). However, the total numbers of cells in blastocysts derived from single‐ and double‐selected embryos produced under moderate polyspermy conditions (60.9 ± 2.5 and 63.5 ± 2.9, respectively) were higher (*p* < .05) than for those produced under high polyspermy conditions (36.9 ± 1.6 and 37.7 ± 3.0, respectively; Table 3).

Under moderate polyspermy conditions, the single‐ and double‐selected embryos showed higher (*p* < .05) competence for blastocyst development (50.2 ± 6.6% and 70.7 ± 11.8%, respectively) than remaining embryos at the time of selection (24.0 ± 5.6%; Table 3). Meanwhile, the rate of development of blastocysts derived from the double‐selected embryos (57.9 ± 4.1%) was significantly higher than that of blastocysts derived from remaining embryos (19.3 ± 4.4%) under high polyspermy conditions (Table 3).

Under high polyspermy conditions, there were no differences in the number of cells per blastocyst derived from single‐, double‐selected, or remaining embryos (36.9 ± 1.6, 37.7 ± 3.0 and 37.5 ± 1.3, respectively). However, the numbers of cells in blastocysts derived from single‐ and double‐selected embryos (60.9 ± 2.5 and 63.5 ± 2.9, respectively) were similar and significantly higher than that of blastocysts derived from remaining embryos (45.1 ± 2.2; Table 3).

### 3.4 Experiment 4

The incidences of nuclear abnormalities in both single‐ and double‐selected embryos produced under moderate polyspermy conditions (20.5 ± 4.2% and 30.1 ± 4.5%, respectively) were significantly (*p* < .05) lower than in those produced under high (38.8 ± 4.7% and 67.6 ± 9.0%, respectively) polyspermy conditions (Table 4).

**TABLE 4:** Comparison of nuclear abnormalities of single‐ and double‐selected porcine embryos produced under moderate and high polyspermy conditions after IVF

Polyspermy condition	No. (%) of single‐selected embryos			No. (%) of double‐selected embryos		
	Total	Normal nuclei	Abnormal nuclei	Total	Normal nuclei	Abnormal nuclei
Moderate	106	83 (79.5 ± 4.2)^a,b^	23 (20.5 ± 4.2)^a,b^	39	27 (69.9 ± 4.5)^a,b^	12 (30.1 ± 4.5)^a,b^
High	105	66 (61.2 ± 4.7)^a,b^	39 (38.8 ± 4.7)^a,b^	28	10 (32.4 ± 9.0)^a,b^	18 (67.6 ± 9.0)^a,b^

Note

Sperm with moderate and high polyspermy were collected from Boars A and B, respectively.

Three to six replications of each sperm line were carried out.

Results are presented as mean percentage ± *SEM*.

^a,b^ Values with different superscripts in the same column are significantly different in each column (*p* < .05).

### 3.5 Experiment 5

The results of chromosome analysis of single‐selected embryos obtained under moderate and high polyspermy conditions are shown in Table 5. The proportion of diploid embryos produced under moderate polyspermy conditions (72.5 ± 9.9%) was significant higher (*p* < .05) than under high polyspermy conditions (16.7 ± 12.5%). Moreover, the incidences of mixoploidy and haploidy in embryos obtained under moderate polyspermy conditions (18.5 ± 9.0% and 2.4 ± 2.4%, respectively) were significantly lower (*p* < .05) than those obtained under high polyspermy conditions (57.6 ± 8.7% and 19.0 ± 5.5%, respectively). Triploidy was found in 4‐cell embryos produced under both moderate and high polyspermy conditions (6.6 ± 4.2% and 6.7 ± 6.7%, respectively).

**TABLE 5:** Comparison of chromosomal abnormalities of single‐selected porcine embryos produced under moderate and high polyspermy conditions after IVF

Polyspermy condition	No. (%) of single‐selected embryos				
	Total	Normal (Diploid)	Mixploidy	Haploid	Triploid
Moderate	31	22 (72.5 ± 9.9)^a,b^	6 (18.5 ± 9.0)^a,b^	1 (2.4 ± 2.4)^a,b^	2 (6.6 ± 4.2)
High	20	3 (16.7 ± 12.5)^a,b^	12 (57.6 ± 8.7)^a,b^	4 (19.0 ± 5.5)^a,b^	1 (6.7 ± 6.7)

Note

Sperm with moderate and high polyspermy were collected from Boars A and B, respectively.

Five to six replications of each sperm line were carried out.

Results are presented as mean percentage ± *SEM*.

^a,b^ Values with different superscripts in the same column are significantly different (*p* < .05).

The proportion of diploid double‐selected embryos obtained under moderate polyspermy conditions (82.4 ± 5.6%) was significantly higher (*p* < .05) than that under high polyspermy conditions (31.0 ± 9.7%; Table 6). Moreover, the incidence of mixoploidy in embryos obtained under moderate polyspermy conditions (17.6 ± 5.6%) was lower (*p* < .05) than that under high polyspermy conditions (53.4 ± 10.3%). Haploid and triploid embryos were not found among the double‐selected embryos produced under moderate polyspermy conditions. Nevertheless, a number of haploid and triploid embryos were produced among the double‐selected embryos in the high polyspermy group (4.2 ± 4.2% and 11.4 ± 1.1%, respectively; Table 6).

**TABLE 6:** Comparison of chromosomal abnormalities of double‐selected porcine embryos produced under moderate and high polyspermy conditions after IVF

Polyspermy condition	No. (%) of double‐selected embryos				
	Total	Normal (Diploid)	Mixploidy	Haploid	Triploid
Moderate	24	20 (82.4 ± 5.6)^a,b^	4 (17.6 ± 5.6)^a,b^	0 (0)	0 (0)^a,b^
High	27	9 (31.0 ± 9.7)^a,b^	15 (53.4 ± 10.3)^a,b^	2 (4.2 ± 4.2)	3 (11.4 ± 1.1)^a,b^

Note

Sperm with moderate and high polyspermy were collected from Boars A and B, respectively.

Three replications of each sperm line were carried out.

Results are presented as mean percentage ± *SEM*.

^a,b^ Values with different superscripts in the same column are significantly different (*p* < .05).

Mixoploid 4‐ and 8‐cell embryos were produced under both moderate and high polyspermy conditions. Among mixoploid embryos, the frequency of (n/2n) was highest among single‐ and double‐selected embryos produced under high polyspermy conditions (57.6 ± 8.7% and 53.4 ± 10.3%, respectively; Tables 5 and 6).

## 4 DISCUSSION

The aim of the present study was to clarify whether selection of 4‐ and 8‐cell stage embryos as single and double selection at specific time points might be an alternative for selection of good‐quality embryos, and how the degree of polyspermy rate might affect the developmental competence, nuclear status, and karyotype of the selected embryos.

First, we set up IVF systems under moderate and high polyspermy conditions using two different lots of frozen epididymal sperm (Boars A and B). These sperm lots had similar motility (40%–50%) after warming; however, when used for IVF at the same concentrations, the rates of polyspermy differed significantly (54.4% vs. 90.4%), whereas the total oocyte penetration rates did not. Consequently, these lots were used for in vitro production of embryos under moderate and high polyspermy conditions, respectively. This result confirmed the variability of polyspermy in pigs among sperm lots frozen by the same method.

When presumptive zygotes produced under moderate and high polyspermy conditions were cultured, we observed a significant decrease in the number of cells per blastocyst under high polyspermy conditions relative to those produced under moderate polyspermy conditions.

Previous studies have shown that selection of good‐quality porcine embryos depends on the timing and patterns of the first cleavage (Dang‐Nguyen et al., 2010; Ulloa Ulloa, Yoshizawa, Komoriya, et al., 2008). On this basis, in the present study we focused on 4‐cell embryos selected at 48 hr after IVF and 8‐cell embryos selected at 79 hr after IVF from 4‐cell embryos as single‐ and double‐selected embryos, respectively. We found that the single‐ and double‐selected embryos had similarly high competence (*p* > .05) for development to the blastocyst stage under both moderate and high polyspermy conditions (Table 3). Moreover, the selected 4‐ and 8‐cell embryos produced under both moderate and high polyspermy conditions developed to blastocysts at significantly higher rates compared with relative to embryos at different stages. Our results are in agreement with previous reports (Dang‐Nguyen et al., 2010; Ulloa Ulloa, Yoshizawa, Komoriya, et al., 2008); 4‐ and 8‐cell embryos chosen at 48 hr and 79 hr after IVF, respectively, had high competence for development to blastocysts even under high polyspermy conditions, suggesting that this morphological approach could be an alternative method for selection of embryos with high developmental ability in pigs. In this study we observed no significant difference between single‐ and double‐selection groups in terms of developmental competence and embryo normality, suggesting that single selection of embryos at the 4‐cell stage at 48 hr after IVF would be sufficient for delineation of embryos with high developmental competence.

However, we also found that the total numbers of cells in blastocysts derived from single‐ and double‐selected embryos produced under moderate polyspermy conditions were significantly higher than in those produced under high polyspermy conditions (Table 3). As mentioned previously, embryos produced under moderate polyspermy conditions had significantly higher developmental competence, in terms of blastocyst rate and total cell number per blastocyst, than those produced under high polyspermy conditions (Table 2). These results confirm previous reports suggesting that high polyspermy conditions likely affect the developmental competence of embryos (Han, Abeydeera, et al., 1999; Somfai et al., 2008). We then performed a more detailed examination of nuclear status and chromosome numbers of single‐ and double‐selected embryos produced under moderate and high polyspermy conditions to determine whether the incidences of nuclear and chromosomal abnormalities were increased in embryos produced under high polyspermy conditions. Nuclear staining with DAPI revealed that the incidences of nuclear abnormalities in both single‐ and double‐selected embryos produced under moderate polyspermy conditions were significantly lower than in those produced under high polyspermy conditions (Table 4). The incidences of nuclear abnormalities in embryos produced under both moderate and high polyspermy conditions were lower than those reported previously in 4‐cell embryos selected at 48 hr after IVF (Wang, Abeydeera, Han, Prather, & Day, 1999).

Similarly, chromosome analysis revealed that the incidences of chromosomal abnormalities were elevated in single‐ and double‐selected embryos produced under high polyspermy condition compared with those in single‐ and double‐selected embryos produced under moderate polyspermy condition (Tables 5 and 6). Significant increases in percentages of the mixoploid 4‐ and 8‐cell embryos produced under high polyspermy condition were also recorded (Tables 5 and 6). Our results confirmed that higher frequency of chromosomal abnormalities is observed in polyspermic embryos compared with monospermic ones (Han, Wang, et al., 1999; Somfai et al., 2008; Ulloa Ulloa, Yoshizawa, Komoriya, et al., 2008). Altogether, the present results suggest that high frequency of nuclear and chromosomal abnormalities in single‐ and double‐selected embryos produced under high polyspermy conditions could be responsible for the reduced quality of the derived blastocysts.

Interestingly, among the embryos produced under moderate polyspermy conditions, we recorded only very low incidences of haploidy and triploidy in single‐selected embryos (Table 5) and found no haploid or triploid embryos among the double‐selected embryos (Table 6). However, a degree of haploidy and triploidy was found among both single‐selected embryos and double‐selected embryos produced under high polyspermy conditions (Tables 5 and 6). A large proportion of single‐ and double‐selected embryos produced under high polyspermy conditions were mixoploid, and approximately half of them showed haploid/diploid (n/2n) mosaicism. It has been suggested that mixoploid and triploid embryos result from polyspermy: haploid/diploid (n/2n) mosaicism occurs when one female and one male pronucleus are close but the other is eccentrically located, whereas triploid embryos are formed when all pronuclei are centrally located (Han, Wang, et al., 1999; reviewed in Funahashi, 2003). The high frequencies of triploid and haploid/diploid (n/2n) mosaic embryos produced under high polyspermy conditions in our study confirm the link between polyspermy and chromosomal ploidy anomalies.

In conclusion, 4‐ and 8‐cell embryos selected at 48 and 79 hr after IVF have been shown to have high competence for development to blastocysts under both moderate and high polyspermy conditions. Such selection of 4‐ and 8‐cell embryos at these respective time points could be used as an alternative option for embryo selection based on morphology even under high polyspermy conditions in porcine IVF. The polyspermy rate affects the frequency of nuclear and chromosomal abnormalities of 4‐ and 8‐cell embryos and the quality of the derived blastocysts.

## CONFLICT OF INTEREST

All authors declare no conflict of interest.
